# Antitumor Effects of Rapamycin in Pancreatic Cancer Cells by Inducing Apoptosis and Autophagy

**DOI:** 10.3390/ijms14010273

**Published:** 2012-12-21

**Authors:** Zhi-Jun Dai, Jie Gao, Xiao-Bin Ma, Hua-Feng Kang, Bao-Feng Wang, Wang-Feng Lu, Shuai Lin, Xi-Jing Wang, Wen-Ying Wu

**Affiliations:** 1 Department of Oncology, the Second Affiliated Hospital, Medical School of Xi’an Jiaotong University, Xi’an 710004, China; E-Mails: binbinmxb@sohu.com (X.-B.M.); kanghf73@yahoo.com.cn (H.-F.K.); wangbf1680@126.com (B.-F.W.); luwangfengsl@126.com (W.-F.L.); linshuai420@stu.xjtu.edu.cn (S.L.); wangxijing@21cn.com (X.-J.W.); 2 Department of Nephrology, the Second Affiliated Hospital, Medical School of Xi’an Jiaotong University, Xi’an 710004, China; E-Mail: gxej_cn@sina.com; 3 Department of Pharmacology, the Second Affiliated Hospital, Medical School of Xi’an Jiaotong University, Xi’an 710004, China

**Keywords:** pancreatic carcinoma, rapamycin, mTOR, anti-tumor, apoptosis, autophagy

## Abstract

Rapamycin (Rapa), an inhibitor of mammalian target of Rapamycin (mTOR), is an immunosuppressive agent that has anti-proliferative effects on some tumors. This study aims to investigate the effects of Rapa suppressing proliferation of pancreatic carcinoma PC-2 cells *in vitro* and its molecular mechanism involved in antitumor activities. MTT assays showed that the inhibition of proliferation of PC-2 cells *in vitro* was in a time- and dose-dependent manner. By using transmission electron microscopy, apoptosis bodies and formation of abundant autophagic vacuoles were observed in PC-2 cells after Rapa treatment. Flow cytometry assays also showed Rapa had a positive effect on apoptosis. MDC staining showed that the fluorescent density was higher and the number of MDC-labeled particles in PC-2 cells was greater in the Rapa treatment group than in the control group. RT-PCR revealed that the expression levels of p53, Bax and Beclin 1 were up-regulated in a dose-dependent manner, indicating that Beclin 1 was involved in Rapa induced autophagy and Rapa induced apoptosis as well as p53 up-regulation in PC-2 cells. The results demonstrated that Rapa could effectively inhibit proliferation and induce apoptosis and autophagy in PC-2 cells.

## 1. Introduction

Pancreatic cancer is the most lethal of the solid tumors and the fourth leading cause of cancer-related death in North America [1,2]. The incidence of pancreatic cancer has been gradually rising, even though the incidence of other common cancers has declined [3]. Eighty to 85% of patients present with locally advanced or metastatic disease that precludes curative resection and have poor prognosis [1,3]. Despite developments in detection and treatment, the five year survival rate of pancreatic cancer is only about 4% [3]. Furthermore, pancreatic cancer responds poorly to most chemotherapeutic agents. In recent years, targeted therapy has become the preferred cancer therapy because of its specificity, targeting inhibition and mild adverse reactions.

Rapamycin (Rapa) is a lipophilic macrolide antibiotic that was initially developed as a fungicide and immunosuppressant [4]. Previous studies have reported that Rapa has anti-proliferative effects on some tumors [5–11]. Rapa acts also as a specific inhibitor of mammalian target of rapamycin (mTOR), a serine/threo-nine kinase that appears to be downstream of the PI3K/Akt signal pathway [12]. mTOR plays a central role in cell growth regulation by integrating signals from growth factors, nutrients, and stress events [13]. It is considered to be a major effector of cell growth and proliferation that controls protein synthesis through a large number of downstream targets [14,15]. The aim of our study was to evaluate the antitumor effect of rapamycin in human pancreatic carcinoma PC-2 cells and to clarify the possible molecular mechanism of rapamycin in inducing apoptosis and autophagy.

## 2. Results and Discussions

### 2.1. Effect of Rapa on Proliferation of PC-2 Cells

Rapa has been shown to have *in vitro* or *in vivo* growth inhibitory effects on a number of cancers including gallbladder cancer, Kaposi sarcoma, laryngeal cancer and prostate cancer [5–11]. Shafer’s research demonstrated that rapamycin potentiated the effects of paclitaxel in endometrial cancer cells through inhibition of cell proliferation, induction of apoptosis and potentially increased polymerization and acetylation of tubulin [16]. Similarly, Rapa could inhibit urothelial carcinoma cell proliferation and enhance the effectiveness of cisplatin [17]. Rapa also has an anti-lymphangiogentic effect and exerts the expected inhibition of lymphatic metastasis [18]. While, in pancreatic cancer, the results were not consistent, in clinic, Rapa was well tolerated. However, no correlation was found between the efficacy of inhibiting mTOR in tumor tissues and anti-tumor effects [19]. RAD001, a rapalog of Rapa, administered as a single-agent, had minimal clinical activity in patients with gemcitabine-refractory, metastatic pancreatic cancer [20]. *In vitro*, Rapa combined with inhibition of the Notch pathway showed a greater efficacy in the treatment of patients with pancreas cancer [21]. Rapa showed dose-dependent antiproliferative effects on pancreatic carcinoma cell lines *in vitro* both alone and in combination with FTY720 [22].

**Figure 1:**
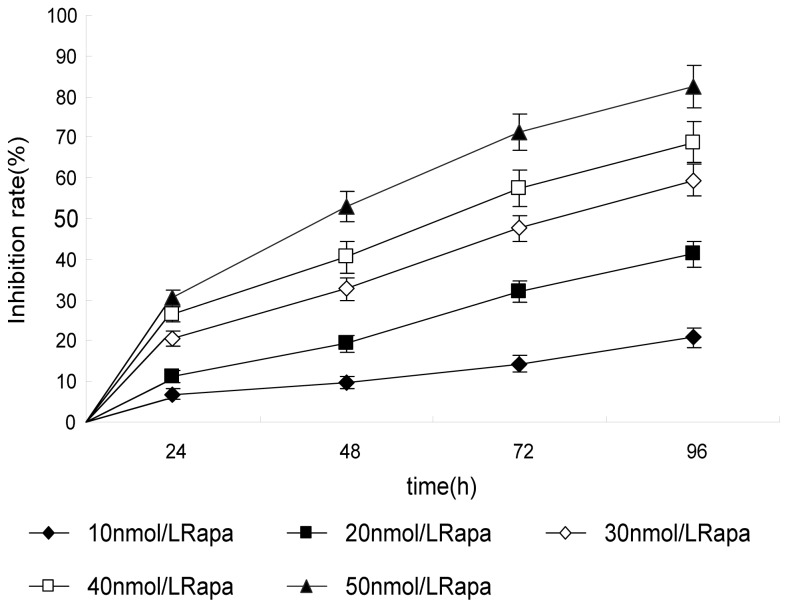
Growth inhibiting effects of Rapamycin (Rapa) on PC-2 cells. PC-2 cells were treated with different concentrations for 0–96 h. Cell viability was determined by MTT method. This assay was performed in triplicate. Dose- and time-dependent inhibition of cell growth could be observed after 96 h (*p* < 0.05, ANOVA analysis).

In this study, PC-2 cells were treated with different doses of Rapa for 0–96 h. MTT assay was used to examine the anti-proliferative effect of Rapa on PC-2 cells. As shown in Figure 1, the inhibitory rate of Rapa on cell growth was as high as (82.5 ± 5.4)%, when the cells were treated for 96 h with high concentrations of Rapa (50 nmol/L). MTT assay showed that Rapa inhibited the proliferation of PC-2 cells, in a dose- and time-dependent manner.

### 2.2. Morphological Observation of Apoptosis and Autophagy of PC-2 Cells Induced by Rapa

Traditionally, apoptosis has been considered to be the predominant type of programmed cell death. Advances in the understanding of autophagy in normal as well as pathological conditions establishes autophagic cell death as an alternative form of cell death, leading to the reclassification of programmed cell death into two types: Type I as apoptotic death and Type II as autophagic death [23,24]. Autophagy is an evolutionarily conserved process of sequestering organelles and long-lived proteins in a double-membrane vesicle, the autophagosomes, for subsequent lysosomal degradation [25].

In normal cells, autophagy contributes to the turnover of long-lived proteins and elimination of damaged or aged organelles, so that to maintain cell homeostasis [26,27]. While under pathological conditions, autophagy is generally considered to play a prosurvival role, recently increasing evidence indicates that autophagy is closely associated with tumors and plays an important role in human tumor suppression [27–29].

**Figure 2:**
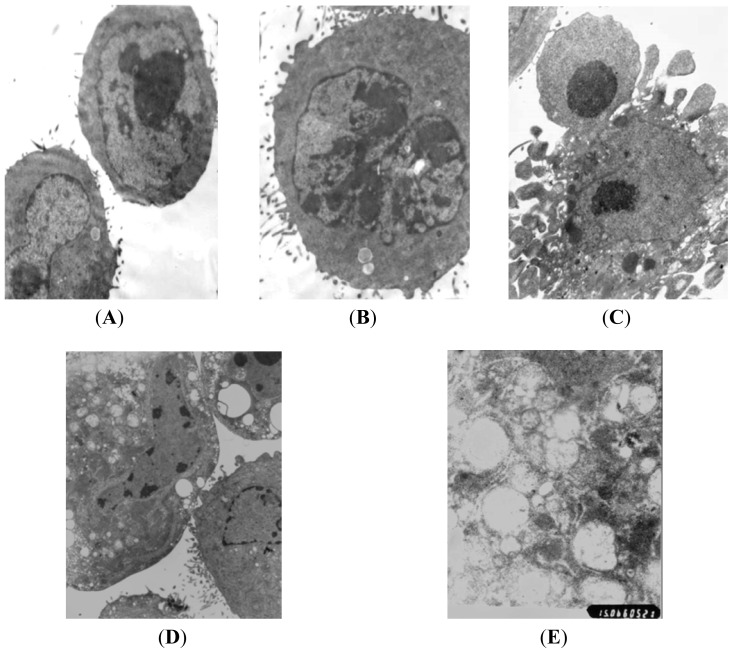
Morphological observation of PC-2 cells by transmission electron microscopy after treatment with Rapa. (**A**) normal PC-2 cells (5000×); (**B**) karyopyknosis and chromatic agglutination (5000×); (**C**) formation of apoptotic body (5000×); (**D**) characteristic ultrastructural morphology of autophagy in PC-2 cells (6000×); (**E**) autophagic vacuoles in PC-2 cells (10,000×).

High resolution transmission electron microscopy showed that normal PC-2 cells were round and regular in shape with chromatin margination in few tumor cells (Figure 2A). After treatment with different doses (10, 30, 50 nmol/L) of Rapa for 48 h, the nuclei showed chromatin pyknosis, and were clustered on the inner border of karyotheca (Figure 2B). The typical morphologies of apoptotic PC-2 cells such as chromatic agglutination and fragmentation of nuclei, chondriosome swelling, formation of apoptotic body, could be observed in the high Rapa dose group (Figure 2C). In the 30 nmol/L Rapa group, characteristic ultrastructural morphology of autophagy was also observed. Abundant autophagic vacuoles sequestrated in cytoplasm and organelles, such as mitochondria and endoplasmic reticulum (Figure 2D,E). The results demonstrated that both autophagy and apoptosis were activated when death of PC-2 cells occurred after Rapa treatment.

### 2.3. Flow Cytometry(FCM) Analysis of Cell Apoptosis Induced by Rapa

mTOR is involved in tumor growth and apoptosis of cancer cells, and can control protein translation [30,31]. In [32–34], Rapa was found to target inhibition of mTOR expression, and could induce apoptosis and inhibit the proliferation of cancer cells. On the other hand, some researchers showed that Rapa alone did not induce apoptosis [16].

**Figure 3:**
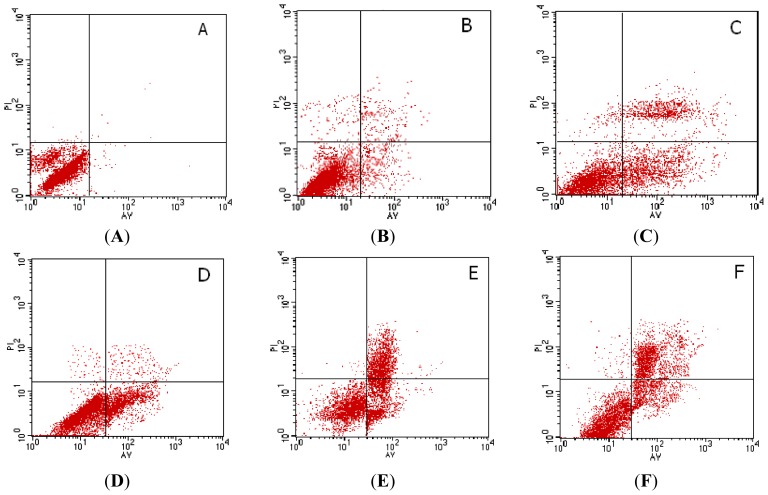

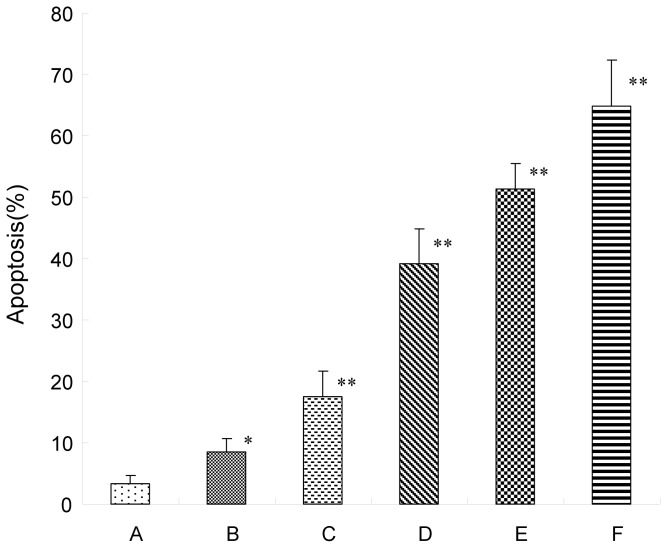
Flow cytometry analysis for PC-2 cells after treatment by Annexin V-FITC and PI staining for apoptosis. (**A**) 0 nmol/L Rapa group; (**B**) 10 nmol/L Rapa group; (**C**) 20 nmol/L Rapa group; (**D**) 30 nmol/L Rapa group; (**E**) 40 nmol/L Rapa group; (**F**) 50 nmol/L Rapa group. * *p* < 0.05, ** *p* < 0.01 *versus* control group.

After treatment with different doses of Rapa for 72 h, apoptosis induction was demonstrated using FCM analysis. Apoptotic cells were differentiated from viable or necrotic ones by combined application of annexin V-FITC and PI. Apoptotic and necrotic cells were distinguished according to annexin V-FITC reactivity and PI exclusion. As shown in Figure 3, in the normal control group, there were almost normal cells and rarely viable apoptotic cells; while in Rapa groups, the rate of apoptotic cells was gradually increased along with increasing concentrations of Rapa. The rate of apoptosis in normal control, 10–50 nmol/L Rapa groups were (8.53 ± 2.14)%, (17.58 ± 4.10)%, (39.24 ± 5.66)%, (51.30 ± 4.12)% and (64.81 ± 7.52)%, respectively. Furthermore, apoptotic cells gradually increased in a dose-dependent manner.

### 2.4. MDC-Labeled Vacuoles in Rapa-Treated PC-2 Cells

In multidrug-resistant v-Ha-ras-transformed NIH3T3 (Ras-NIH3T3/Mdr) cells, Eum and Lee [35] demonstrated that rapamycin-induced cell death may result from two different mechanisms. At high rapamycin concentrations (≥100 nM), cell death may occur via an autophagy-dependent pathway, whereas at lower concentrations (≤10 nM), cell death may occur after G1-phase cell cycle arrest.

**Figure 4:**
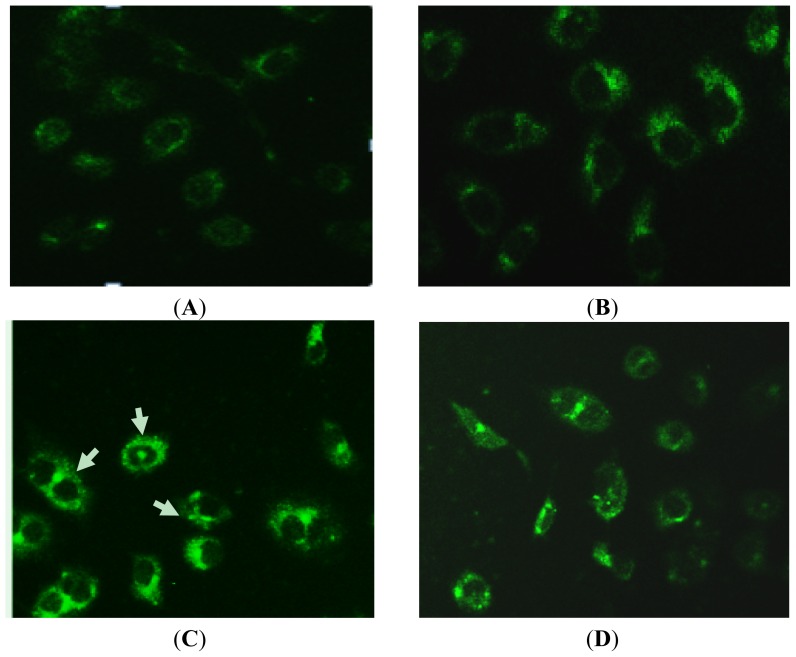
MDC-labeled autophagic vacuoles in PC-2 cells by fluorescence microscope after treatment with Rapa. Autophagic vacuoles were labeled with 0.05 mmol/L MDC in phosphatebuffered saline (PBS) at 37 °C for 10 min. (**A**) 0 nmol/L Rapa group; (**B**) 10 nmol/L Rapa group; (**C**) 50 nmol/L Rapa group; (**D**) 50 nmol/L Rapa + 3-MA group (200×).

To investigate the inducing autophagy effect of Rapa in PC-2 cells, we used a fluorescence microscope with monodansylcadaverine (MDC) staining. MDC is a specific marker for autophagic vacuoles [36]. When the cells were viewed under a fluorescence microscope, MDC-labeled autophagic vacuoles appeared as distinct dot like structures distributing in cytoplasm or in perinuclear. 3-methyladenine (3-MA) was a specific autophagic inhibitor. As shown in Figure 4, the fluorescent density and MDC-labeled particles of PC-2 cells were higher in Rapa treatment group than in control group, indicating that Rapa induces formation of MDC-labeled vacuoles. Fewer autophagic vacuoles were observed in combined 3-MA and Rapa treatment group when 3-MA was added before Rapa treatment, showing that 3-MA exerted its inhibitory effects on Rapa-treated autophagy. The number of MDC-labeled particles in PC-2 cells was significantly fewer in combined 50 nmol/L Rapa and 3-MA treatment group than in 50 nmol/L Rapa treatment group. The results indicated that autophagy was activated when Rapa-induced death of PC-2 cells occurs.

### 2.5. Expression of mTOR, p53, Bax and Beclin 1 Detected by Reverse-Transcription PCR(RT-PCR)

**Figure 5:**
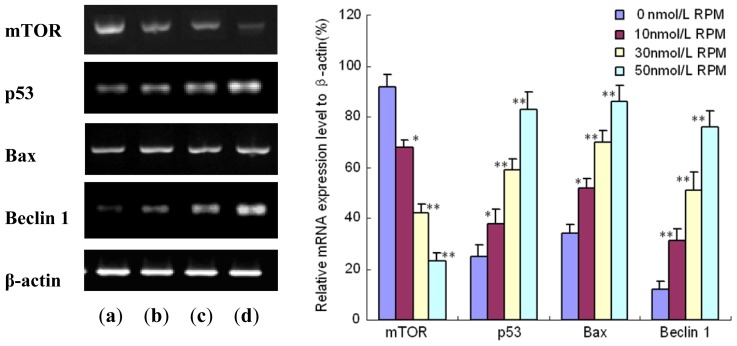
The mRNA expression of mTOR, p53, Bax and Beclin 1 in PC-2 cells treated with different concentrations of Rapa: After treatment with different doses of Rapa for 24 h, mRNA level was detected by semi-quantitive RT-PCR analysis. This assay was done quintuplicate. Values represent means ± standard deviations and were determined using the Student’s t-test. * *p* < 0.05 and ** *p* < 0.01 *versus* 0 nmol/L Rapa group. (**a**) 0 nmol/L Rapa group; (**b**) 10 nmol/L Rapa group; (**c**) 30 nmol/L Rapa group; (**d**) 50 nmol/L Rapa group.

As we know, p53 plays a pivotal role in apoptosis. In addition, p53 is currently being extensively investigated as a promising strategy for highly specific anticancer therapy in chemotherapeutics therapy [37]. Miyake *et al.* [38] observed that Rapa could induce p53-independent apoptosis through the mitochondrial pathway in non-small cell lung cancer cells. To investigate the mechanisms underlying the apoptosis induced by Rapa, the mRNA expression level of p53 and Bax gene in PC-2 cells treated with Rapa was measured by RT-PCR. As shown in Figure 5, p53 mRNA expression in PC-2 cells was up-regulated in a dose-dependent manner. Bax was the first member of bcl-2 group shown to be induced by p53 [39]. Bax mediated more than about 50% of the p53-dependent cell apoptosis [40]. At the same time, Rapa gradually increased the Bax mRNA expression level when the concentration of Rapa was increased. The results demonstrated that Rapa induced apoptosis as well as p53 up-regulation in PC-2 cells.

Beclin 1, a mammalian orthologue of the yeast Apg6/Vps30 gene, is the first identified mammalian gene to induce autophagy [28]. Beclin1 functions in autophagy as part of class III phosphatidylinositol 3-kinase (PI3k) complex, which is necessary for the formation of autophagosome during the autophagic sequestration process [41,42]. In this study, the mRNA expression level of Beclin 1 in PC-2 cells was measured to elucidate the mechanism of autophagy induced by Rapa. RT-PCR showed that Rapa activated the Beclin 1 gene expression in a dose-dependent manner (Figure 5). In other words, the Beclin 1 mRNA expression level steadily increased with the concentration of Rapa.

## 3. Experimental Section

### 3.1. Reagents

Fetal bovine serum (Gibco, USA); RPMI1640 medium (Gibco, USA); 3-(4,5)-dimethylthiahiazo (-z-y1)-3,5-diphenyte- trazoliumromide (MTT) (Gibco, USA); annexin V-FITC/PI apoptosis detection kit (Becon Dickinson Facsalibur, USA); RT-PCR kit (Ampliqon, Denmark); Trizol (Invitrogen, USA); mTOR monoclonal antibody (Santa Cruz Biotechnology, USA); Rapamycin (Rapa) (Sigma, USA); monodansylcadaverine (MDC) (Sigma, USA); 3-methyladenine (3-MA) (Sigma, USA).

### 3.2. Cell Line and Cell Culture

Human pancreatic cancer cell line, PC-2 was obtained from Shanghai Institute of Cell Biology, Chinese Academy of Sciences (Shanghai, China). Cells were cultured in RPMI 1640 maximal medium containing 10% inactivated fetal bovine serum (56 °C, 30min), 1 × 10^5^ U/L penicillin and 100 mg/L streptomycin in a humidified atmosphere with 5% CO_2_ incubator at 37 °C.

### 3.3. MTT Assay for the Proliferation of Pancreatic Cancer Cells

Viability of PC-2 Cells was assessed using MTT dye reduction assay (Sigma, USA), which was conducted as described previously [43]. Cells were seeded in a 96-well plate at a density of 1 × 10^4^ cells/well, cultured for 12 h, then treated with different concentration (10, 20, 30, 40, 50 μmol/L) Rapa for 0–96 h. At the end of the treatment, MTT, 50 μg/10 μL, was added and the cells were incubated for another 4 h. Dimethylsufloxide (DMSO; 200 μL) was added to each well after removal of the supernatant. After shaking the plate for 10 min, cell viability was assessed by measuring the absorbance at 490 nm using an Enzyme-labeling instrument (EX-800 type); all measurements were performed four times. Cell growth curve was completed using time as the abscissa and A value (mean ± SD) as the ordinate.

### 3.4. Detection of Morphological Change by Transmission Electron Microscope

Uranyl acetate and lead citrate staining of cells were performed to detect morphological changes. Briefly, adherent PC-2 cells were treated with 50 nmol/L Rapa for 48 h. After treatment, the treated cells were digested with pancreatin and fixed with 3% glutaraldehyde precooled in 4 °C for 2 h. To make ultra-thin sections of copper, cells were washed with phoisphate-buffered salein (PBS) once, fixed with 1% osmic acid for 1 h, dehydrated by acetone and embedded in epoxide resin. After staining with uranyl acetate and lead citrate, the sections were examined by a Hitachi-800 transmission electron microscope [44].

### 3.5. Apoptosis Detection by FCM

Apoptotic cells were differentiated from viable or necrotic ones by combined application of annexin V-FITC and propidium iodide (PI) (BD Biosciences Clontech, USA) [45]. The samples were washed twice and adjusted to a concentration of 1 × 10^6^ cells/mL with 4 °C PBS. The Falcon tubes (12 mm × 75 mm, polystyrene round-bottom) were used in this experiment, 100 μL of suspensions was added to each labeled tube, 10 μL of annexin V-FITC and 10 μL PI(20 μg/mL) were added into the labeled tube, incubated for at least 20 min at room temperature in the dark, then 400 μL of PBS binding buffer was added to each tube without washing and analyzed using FCM analysis (BD Biosciences Clontech, USA) as soon as possible (within 30 min). This assay was done quintuplicate.

### 3.6. MDC Staining of Autophagic Vacuoles

MDC staining of autophagic vacuoles was performed for autophagy analysis as previously described [46]. PC-2 cells were divided into control group, 3-MA treatment group, Rapa treatment group, and combined 3-MA and Rapa treatment group. The cells were incubated for 48 h on coverslips. Autophagic vacuoles were labeled with 0.05 mmol/L MDC in PBS at 37 °C for 10 min. And then, the cells were washed three times with PBS. Autophagic vacuoles in PC-2 cells were observed under a fluorescence microscope (Olympus, BX-60, Japan). Fluorescence intensity of MDC was measured at an excitation wavelength of 380 nm, emission wavelength of 530 nm.

### 3.7. Semi-Quantitative Reverse Transcription Polymerase Chain Reaction (RT-PCR) Assay

PC-2 cells were seeded in 6cm culture capsules and treated with concentration gradient Rapa (0, 10, 20, 30, 40, 50 μmol/L) separately for 8h. Each group contained 3 culture capsules. As previously described [47], cells collected at specified time were used to extract total RNA using the Trizol reagent following the manufacturer’s instructions. 1 μg RNA synthetized cDNA through reverse transcriptase undergo listed below condition: 70 °C 5 min, 42 °C extended for 60 min, 95 °C enzyme inactivated for 3 min and 4 °C terminated reaction; Synthetical cDNA as template to carry out polymerase chain reaction. mTOR primer sequence (Invitrogen CO): 5′-CGCTGTCATCCCTTTATCG-3′ (sense) and 5′-ATGCTCAAACACCTCCACC-3′ (anti-sense), amplification fragment was 218bp, renaturation temperature was 55 °C (cycling 25 times). wt p53 primer sequence (Invitrogen CO): 5′-ACACCTG GATCGTTACTCGGCTTGTC-3′ (sense) and 5′-GCTAGAAAGTCAACATCAGT CTAGG-3′ (anti-sense), amplification fragment was 168bp. Bax primer sequence (Invitrogen CO): 5′-CCAGGATC GAGCAGGGAGG-3′ (sense) and 5′-GAGCGAGGCGGTGAGGACT-3′ (anti-sense), amplification fragment was 95bp. Beclin 1 primer sequence (Invitrogen CO): 5′-CCTCG TGCTGAGGGATGGAA-3′ (sense) and 5′-GCCGTAGCATTGCCTGGGCTG-3′ (anti-sense), amplification fragment was 192bp. β-actin primer sequence was 5′-GTTGCGTTACACCCTTTCTTG-3′ (sense), 5′-TGCTGTCACCTT CACCGTTC-3′ (anti-sense), amplification fragment was 133 bp. Renaturation temperature was 55 °C (cycling 20–25 times). Amplification condition was below: pre-denaturized for 3 min at 95 °C, denaturized for 30s at 95 °C, renaturated for 30s at 55 °C and extended for 30s at 72 °C. PCR product was detected on agarose gel electrophoresis and ethidium bromide imaging system was used to make density index analysis. The expression intensity of destination gene mRNA was denoted with the ratio of the photodensity of the RT-PCR products of destination gene and β-actin.

### 3.8. Statistical Analysis

All data were expressed by mean ± S.E.M. Statistical analyses were performed using SPSS 13.0 for Windows software. ANOVA (one-way analysis of variance) and Student’s *t*-test were used to analyze statistical differences between groups under different conditions. *p*-value <0.05 was considered statistically significant.

## 4. Conclusions

In conclusion, MTT assays showed that Rapa could inhibit the proliferation of PC-2 cells *in vitro* in a time- and dose-dependent manner. Both autophagy and apoptosis were activated when death of PC-2 cells occurred after Rapa treatment. RT-PCR results indicated that Beclin 1 was involved in Rapa induced autophagy and Rapa induced apoptosis as well as p53 up-regulation in PC-2 cells. However, further studies are necessary to clarify the detailed mechanism involved in the antitumor effects of Rapa.
